# Cardiovascular parameters on computed tomography are independently associated with in-hospital complications and outcomes in level-1 trauma patients

**DOI:** 10.1007/s00068-022-02168-7

**Published:** 2022-11-27

**Authors:** Tim Kobes, Arthur A. R. Sweet, IJsbrand T. Klip, Roderick M. Houwert, Wouter B. Veldhuis, Luke P. H. Leenen, Pim A. de Jong, Mark C. P. M. van Baal

**Affiliations:** 1 Department of Surgery, University Medical Center Utrecht, PO Box 85500, 3508 GA Utrecht, The Netherlands; 2 Department of Radiology, University Medical Center Utrecht, PO Box 85500, 3508 GA Utrecht, The Netherlands; 3 Department of Cardiology, Antonius Hospital, PO Box 20000, 8600 BA Sneek, The Netherlands; 4 Department of Cardiology, University Medical Center Groningen, PO Box 30001, 9700 RB Groningen, The Netherlands

**Keywords:** Opportunistic screening, Trauma patients, In-hospital complications, Coronary artery calcification, Abdominal aorta calcification, Pulmonary emphysema

## Abstract

**Background:**

In-hospital complications after trauma may result in prolonged stays, higher costs, and adverse functional outcomes. Among reported risk factors for complications are pre-existing cardiopulmonary comorbidities. Objective and quick evaluation of cardiovascular risk would be beneficial for risk assessment in trauma patients. Studies in non-trauma patients suggested an independent association between cardiovascular abnormalities visible on routine computed tomography (CT) imaging and outcomes. However, whether this applies to trauma patients is unknown.

**Purpose:**

To assess the association between cardiopulmonary abnormalities visible on routine CT images and the development of in-hospital complications in patients in a level-1 trauma center.

**Methods:**

All trauma patients aged 16 years or older with CT imaging of the abdomen, thorax, or spine and admitted to the UMC Utrecht in 2017 were included. Patients with an active infection upon admission or severe neurological trauma were excluded. Routine trauma CT images were analyzed for visible abnormalities: pulmonary emphysema, coronary artery calcifications, and abdominal aorta calcification severity. Drug-treated complications were scored. The discharge condition was measured on the Glasgow Outcome Scale.

**Results:**

In total, 433 patients (median age 50 years, 67% male, 89% ASA 1–2) were analyzed. Median Injury Severity Score and Glasgow Coma Scale score were 9 and 15, respectively. Seventy-six patients suffered from at least one complication, mostly pneumonia (*n* = 39, 9%) or delirium (*n* = 19, 4%). Left main coronary artery calcification was independently associated with the development of any complication (OR 3.9, 95% CI 1.7–8.9). An increasing number of calcified coronary arteries showed a trend toward an association with complications (*p* = 0.07) and was significantly associated with an adverse discharge condition (*p* = 0.02). Pulmonary emphysema and aortic calcifications were not associated with complications.

**Conclusion:**

Coronary artery calcification, visible on routine CT imaging, is independently associated with in-hospital complications and an adverse discharge condition in level-1 trauma patients. The findings of this study may help to identify trauma patients quickly and objectively at risk for complications in an early stage without performing additional diagnostics or interventions.

**Supplementary Information:**

The online version contains supplementary material available at 10.1007/s00068-022-02168-7.

## Background

In the Netherlands, over 19,000 patients were admitted to a level-1 trauma center in 2017 [1]. In-hospital complication rates in level-1 trauma patients vary between 4.2 and 24.7% and are associated with prolonged stay, higher costs, and increased mortality [2–5]. In addition, a complicated hospital stay is associated with worse long-term functional outcomes, such as decreased mobility and social activity [6]. Infectious complications are the most prevalent, associated with increased mortality, and considered quality indicators [5, 7–9].

Previous studies in trauma patients have shown risk factors for developing complications, including age, sex, injury severity, and pre-existing cardiopulmonary comorbidities [10–15]. Most of these studies were based on medical history data to assess comorbidities. Risk factors for cardiovascular disease include age, smoking, high body mass index, diabetes mellitus, hyperlipidemia, hypertension, and renal disease [16–18]. Some risk factors can be easily obtained from medical history or through anamnesis. However, performing extensive anamnesis in the trauma bay can be challenging, and, for instance, blood pressure or renal function can be affected due to trauma. An objective and quick evaluation of cardiovascular disease in trauma patients would be helpful in risk assessment. Recent literature showed that cardiopulmonary abnormalities on routinely performed computed tomography (CT) images may independently predict short- and long-term outcomes in non-trauma patients [19–21]. In these studies, chest CTs were performed for various clinical purposes, while, to our knowledge, no such studies have been performed in trauma populations yet. CT images are routinely taken in trauma patients admitted to the trauma bay with suspected injuries of the chest or abdomen. These CT images can show abnormalities, suspectable for cardiopulmonary disease, that might predict complications.

Identifying patients at increased risk of complications might help prevent complications, as prophylactic treatment and additional tests could be started early. Yet, it remains unknown whether CT visible cardiopulmonary abnormalities can predict complications after trauma. Therefore, this study assessed the association between cardiopulmonary abnormalities visible on routine CT images and the development of in-hospital complications and discharge outcomes in patients in a level-1 trauma center.

## Methods

### Study design and participants

A retrospective cohort study was performed at the University Medical Center Utrecht (UMCU), a level-1 trauma center in the Netherlands. The medical ethical committee of the UMCU approved this study and waived the need for consent (protocol number 20-599/C).

Trauma patients aged 16 years or older and hospitalized between January 1st, 2017, and December 31st, 2017, were identified through the local trauma registry. All consecutive patients who underwent CT imaging of the thorax, abdomen, aorta, thoracic spine, or lumbar spine within seven days of admission were included. At least one of the predefined radiologic parameters had to be assessed on these CT images. Exclusion criteria were an active infection upon admission, active outpatient antibiotic treatment or prophylaxis upon admission, transfer to another hospital during hospital admission, or treatment for more than 24 h in another hospital before transportation to the UMCU. Patients who suffered from severe brain injury—defined as an Abbreviated Injury Scale (AIS) score of the head of 3 or higher—were excluded as well since these injuries almost exclusively determine these patients' prognosis [22, 23].

### Radiologic imaging and technique

All CT imaging was routinely performed using either 64- or 256-detector row scanners from Philips Medical Systems (Cleveland, OH, USA). All CT images were viewed using PACS IDS7 21.1.2 (SECTRA), and 0.9 and 5.0-mm slices were available. An intravenous contrast agent was administered in abdominal or thoracic CT imaging during trauma screening or angiographic scans of the aorta. In case an intravenous contrast agent was administered (89.6%, *n* = 388), the split bolus technique was used to simultaneously assess the portal and the arterial phase.

### Radiologic parameter definitions and assessment

Thoracic CT images were analyzed for the presence of coronary artery calcifications and pulmonary emphysema, and abdominal CT images for the presence of calcification in the abdominal aorta and its degree of severity when present.

The coronary arteries and abdominal aorta were assessed on CT images in the bone and soft tissue settings; only calcified plaques were identified. The presence of coronary calcifications was assessed for the left main coronary artery (LM), left anterior descending (LAD) artery, left circumflex (LCx) artery, and right coronary artery (RCA) separately. The extent of calcification of the abdominal aorta was determined based on its circularity and divided per 90 degrees of circularity (i.e., possible scoring of 0 up to 4) as previously described by Hendriks et al. [24]. Pulmonary emphysema was determined using the pulmonary setting and considered present in case centrilobular emphysema was observed. Only centrilobular emphysema was considered in this study, as this specific entity has been associated with smoking, in contrast to other entities that might have more genetic or uncertain etiologies [25].

Two medical doctors (TK, AS) performed the radiologic assessment and discussed borderline cases with a senior staff radiologist with over 13 years of experience in thoracic and abdominal CT imaging evaluation (PdJ). The investigators were blinded to the outcomes during the radiologic analysis. Both investigators and the senior staff radiologist assessed sixty randomly selected cases to evaluate interobserver variability. The results of the interobserver variability analysis were very good, with Cohen's kappas of 0.77 or higher (Supplemental Table 1).

The inclusion criterion was any relevant CT imaging of the chest or abdomen. Therefore, it was unfeasible to assess all radiologic parameters in every patient. For example, when only abdominal imaging was performed, assessment of the coronary arteries was automatically excluded. If one or more radiologic parameters were unavailable, only these parameters were excluded from the analysis, while the available parameters were included. The number of incomplete examinations was stated in the tables as ‘missing’. No imputation methods were performed since we aimed to assess routine CT imaging. The results may be applied easily in patients with routine imaging; imputation methods would not increase the implementation of this opportunistic screening.

### Outcomes

The medical health records were screened for outcomes. The primary outcome was the development of one or more complications, cumulated from all separately scored complications: infectious complications treated with antibiotics (i.e., pneumonia, urinary tract infection, wound infection, other infectious complications) and pharmacologically managed delirium (e.g., antipsychotics or benzodiazepines). Diagnosis and treatment of complications were part of regular care; no additional clinical criteria were used.

Secondary outcomes were intensive care unit (ICU) admission, hospital length of stay (HLOS) and ICU length of stay, days on mechanical ventilation, and the discharge condition measured using the Glasgow Outcome Scale (GOS) score. The local trauma registry provided secondary outcomes.

Follow-up lasted until hospital discharge or in-hospital death. No censoring for competing risks was performed; patients that died during hospital stay (1.8%, *n* = 8) were included in the analyses.

### Variables

The local trauma registry provided age, gender, pre-trauma American Society of Anesthesiologists (ASA) classification, Injury Severity Score (ISS), Glasgow Coma Scale (GCS) score, AIS codes, and mechanism of injury. Radiologic images were obtained from the medical records manually.

### Statistical analysis

All statistical analyses were performed using RStudio 1.4.1717 for Mac (© The R Foundation for Statistical Computing, 2019) with additional packages. The GOS score was separated into a favorable (i.e., good recovery or light disability) or an adverse discharge condition (i.e., severe disability, persistent vegetative state, or mortality) for further analysis. Coronary calcifications of the LAD, LCx, and RCA were made ordinal into one-, two-, or three-vessel coronary artery calcifications—as inspired by coronary artery disease; the LM was separately considered since it is considered a more severe condition associated with multivessel coronary artery disease [26, 27]. ASA classification was dichotomized into severe (ASA 3–4) or no severe comorbidities (ASA 1–2).

Normally distributed variables were described using the mean and standard deviation (SD), non-normally distributed or ordinal variables using the median and interquartile range (IQR), and categorical variables using the proportions.

The association between radiologic abnormalities and several outcomes—complications, infectious complications, pneumonia, delirium, an adverse discharge condition, and HLOS—was assessed. HLOS was log-transformed before analyses. Results were back-transformed before being presented.

Logistic regression analysis was used for the dichotomous and ordinal outcomes; results were presented with the odds ratio (OR) and 95% confidence interval (95% CI). Linear regression analysis was used for HLOS; results were presented with the beta-coefficient and 95% CI. Regression analyses were performed crude and adjusted for covariates. Age, sex, ASA classification, and ISS were chosen as covariates for each separate radiologic abnormality parameter based on clinical experience and previous literature [10–15]. Furthermore, age is a risk factor for the radiologic parameters [16–18, 28].

The *p* for trend was calculated for the number of coronary arteries and the degree of abdominal aorta calcification. In crude analysis, the Cochran-Armitage test was used for dichotomous or ordinal variables; linear regression was used for HLOS. In multivariable analysis, the additional value of the ordinal value to the model was assessed using the likelihood ratio test for all outcomes.

Outcomes were considered statistically significant with a *p* value < 0.05 despite the number of evaluated associations. No correction for multiple testing was made because of the study's explorative nature.

## Results

During the study period, 658 patients underwent a CT scan of the abdomen, thorax, lumbar or thoracic spine, or aorta within seven days of hospital admission. The study cohort consisted of 433 patients after the exclusion of patients due to infection (*n* = 10) or administration of antibiotics (*n* = 12), transfer to another hospital or treatment elsewhere for > 24 h (*n* = 49), an AIS of the head of 3 or higher (*n* = 153), or insufficient information after transfer to the UMCU (*n* = 1).

In the study cohort, the median age was 50 years (IQR 30–65), 67.0% of patients were male (*n* = 290), 33.0% were female (*n* = 143), and most patients were in good health prior to trauma (88.7% ASA classification 1–2). The most prevalent mechanisms of injury were motorized vehicle crashes (35.3%), low-energetic falls (21.7%), high-energetic falls (15.5%), and bicycle accidents (15.2%). The median ISS was 9 (IQR 5–14), and the median GCS score at admission was 15 (IQR 14–15). The scan was made within one day of emergency department admission in 98.6% (*n* = 427) of patients. All cohort characteristics are summarized in Table 1, and differences between subgroups in Supplemental Table 2.

**Table 1:** Characteristics of trauma patients who underwent CT imaging of the thorax or abdomen in a level-1 trauma center (*n* = 433)

Age (median [IQR])	50 [30–65]
Sex	
Male	290 (67.0%)
Female	143 (33.0%)
ASA classification (median [IQR])	2 [1–2]
1–2	384 (88.7%)
3–4	49 (11.3%)
Mechanism of injury	
MVC	153 (35.3%)
Bicycle	66 (15.2%)
High-energetic fall	67 (15.5%)
Low-energetic fall	94 (21.7%)
Other	53 (12.2%)
ISS (median [IQR])	9 [5–14]
< 16	327 (75.5%)
16–25	75 (17.3%)
> 25	31 (7.2%)
GCS score (median [IQR])	15 [14–15]
AIS score per body region (median [IQR])	
Head	1 [0–1]
Thorax	1 [0–3]
Abdomen	0 [0–0]
Spine	0 [0–2]
Upper extremity	0 [0–1]
Lower extremity	0 [0–2]
Days to scan	
0 days	427 (98.6%)
1 day	4 (0.9%)
4–5 days	2 (0.5%)

*AIS* Abbreviated Injury Scale, *ASA* American Society of Anesthesiologists, *CT* computed tomography, *GCS* Glasgow Coma Scale, *IQR* interquartile range, *ISS* Injury Severity Score, *MVC* motor vehicle collision

Seventy-six patients (17.6%) suffered from one or more complications during their hospital stay. The most prevalent complications were pneumonia in 39 patients (9.0%) and delirium in 19 patients (4.4%). Twenty-seven patients (6.2%) had an adverse GOS score, of whom eight patients (1.8%) died during hospital stay. One death was due to pneumonia. The other deaths were direct sequelae of traumatic injuries (Table 2).

**Table 2:** Summary of outcomes in trauma patients who underwent CT imaging of the thorax or abdomen in a level-1 trauma center (*n* = 433)

Complication(s)	76 (17.6%)
Pneumonia	39 (9.0%)
Urinary tract infection	13 (3.0%)
Wound infection	11 (2.5%)
Other infectious complications	11 (2.5%)
Delirium	19 (4.4%)
Adverse GOS score	27 (6.2%)
Mortality	8 (1.8%)
Hospital length of stay (median [IQR])	5 [2–10]
ICU admission	70 (16.2%)
ICU length of stay (median [IQR])^a^	3 [2–6]
Days on mechanical ventilation (median [IQR])^b^	1 [1–3]

*CT* computed tomography, *GOS* Glasgow Outcome Scale, *HLOS* hospital length of stay, *ICU* intensive care unit, *IQR* interquartile range

^a^For ICU admitted patients; ^b^For patients on mechanical ventilation

Table 3 shows the associations between all considered radiologic predictors and the development of one or more complications. Supplemental Tables 3–7 summarize the regression analysis results for the specific complications, HLOS, and an adverse discharge condition.

**Table 3:** The association of scored radiologic parameters and complications in trauma patients who underwent CT imaging of the thorax or abdomen in a level-1 trauma center, before and after adjustment for covariates. Logistic regression analysis was used

Variable	Score	No complication (*n* = 357)	Complication (*n* = 76)	Crude		Adjusted^a^	
				OR (95% CI)	*p* value	OR (95% CI)	*p* value
Left main	0	321	54	*Reference*		*Reference*	
	1	24	18	4.46 (2.25–8.75)	< 0.001	3.86 (1.68–8.91)	< 0.001
	Missing	12	4				
Number of calcified coronary arteries	0	252	42	*Reference*		*Reference*	
	1	48	6	0.75 (0.27–1.74)	0.54	0.46 (0.14–1.29)	0.16
	2	21	11	3.14 (1.37–6.89)	< 0.001	1.75 (0.60–4.89)	0.29
	3	24	13	3.25 (1.50–6.81)	< 0.001	1.86 (0.70–4.93)	0.21
	Missing	12	4	*p* for trend^b^	< 0.001	*p* for trend^c^	0.07
Abdominal aorta	0	201	27	*Reference*		*Reference*	
	1	63	13	1.54 (0.73–3.11)	0.24	0.95 (0.36–2.44)	0.92
	2	35	9	1.91 (0.79–4.29)	0.13	0.69 (0.21–2.16)	0.53
	3	18	7	2.90 (1.05–7.34)	0.03	1.35 (0.35–5.02)	0.66
	4	30	20	4.96 (2.47–9.96)	< 0.001	1.96 (0.62–6.38)	0.26
	Missing	10	0	*p* for trend^b^	< 0.001	*p* for trend^c^	0.42
Pulmonary emphysema	0	322	63	*Reference*		*Reference*	
	1	24	10	2.13 (0.93–4.56)	0.06	1.42 (0.53–3.51)	0.46
	Missing	11	3				

*ASA* American Society of Anesthesiologists, *CT* computed tomography, *CI* confidence interval, *ISS* Injury Severity Score, *OR* odds ratio

^a^Separately adjusted for age, gender, ASA classification, and ISS

^b^Using the Cochran–Armitage test

^c^Using the likelihood ratio test in model with and without the variable

Coronary artery calcifications showed associations with several outcomes. Both LM calcification (OR 4.5, 95% CI 2.3–8.8) and the number of coronary arteries with calcifications (*p* for trend < 0.001) were associated with complications. After adjustment for covariates, calcification of the LM (OR 3.9, 95% CI 1.7–8.9) was still significantly associated with the development of complications; coronary artery calcifications still showed strong signals of an association with complications, yet without significantly adding value to the multivariable model (*p* likelihood = 0.07). LM calcifications increased the risk of developing an infectious complication (OR 3.9, 95% CI 1.6–9.2) and delirium (OR 3.3, 95% CI 1.0–11.1). The association between LM calcification and HLOS was not statistically significant (β coefficient 1.3, *p* = 0.07). Furthermore, the number of calcified coronary arteries significantly contributed to predicting an adverse discharge condition (*p* likelihood = 0.02).

The degree of the abdominal aorta calcification was significantly associated with complications (*p* for trend < 0.001), pneumonia (*p* = 0.01), delirium, infectious complications, and HLOS (all *p* < 0.001) in crude analysis but with none of the outcomes after adjustment.

Pulmonary emphysema showed no association with the development of complications in the crude or adjusted logistic regression analysis. Pulmonary emphysema was only associated with developing an infectious complication in crude analysis (*p* = 0.02), which was no longer apparent in the multivariable model.

## Discussion

The current study assessed whether cardiopulmonary abnormalities visible on routine trauma CT predict in-hospital complications in Dutch level-1 trauma patients. To our knowledge, this is the first study that considers associations between these radiologic parameters and complications in trauma patients. Calcification in the left main coronary artery was independently associated with developing any in-hospital complication, infectious complications, and delirium. An increasing number of calcified coronary arteries showed a trend towards an independent association with in-hospital complications and was significantly associated with an adverse discharge condition. Pulmonary emphysema and calcification of the abdominal aorta were not independently associated with complications or outcomes.

Previous studies in trauma patients that investigated comorbidities as a risk factor for complications frequently used either ASA classification or the Charlson Comorbidity Index (CCI) to score the severity of comorbidities [11, 12, 14, 15]. A study by Lima et al. of elderly hip fracture patients found that the CCI score was not independently associated with in-hospital delirium (*p* ≥ 0.862), but heart disease and chronic obstructive pulmonary disease were [14]. When considering 30-day complications in elderly hip fracture patients, CCI is significantly associated with complications, but ASA classification appears to be a better predictor [29]. In the current study, calcification of the LM was still associated with complications, even after adjustment for ASA classification [12]. Hence, a quick assessment of the LM might provide additional predictive value, besides other proven predictors, to identify trauma patients at risk for in-hospital complications.

The findings in the current study correspond with previous studies of non-trauma patients. Borggreve et al. found that coronary artery calcification is independently associated with anastomotic leakage in esophagectomy patients (OR 2.3, 95% CI 1.3–4.1) [19]. Their multivariable analysis corrected for age, body-mass index, comorbidities (i.e., cardiovascular, chronic obstructive pulmonary disease, diabetes mellitus), and a history of smoking. Coronary artery calcifications were considered an ordinal variable, with a score ranging from 0 to 2 (with a higher score representing more severe calcifications), though the number of affected arteries was not considered. The present study of trauma patients reported similar odds ratios for complication development when considering the LM.

Coronary artery disease is often concomitant with other comorbidities, such as diabetes, hypertension, and obesity [16–18]. Moreover, patients with LM coronary artery disease have poorer outcomes than patients without an affected LM [30]. Therefore, one could speculate that LM calcification is indicative of a higher (pre-trauma) risk profile with decreased resilience and subsequently with in-hospital complications in trauma patients.

Cohen's kappas were very good for all measured radiologic predictors. This coefficient was calculated for sixty randomly selected patients, in whom both the senior staff radiologist and the scoring investigators without any significant radiologic experience scored the considered radiologic abnormalities. Therefore, readers with limited experience could perform a quick but proper risk assessment in the emergency department. The reliability and convenience of the described cardiovascular and pulmonary abnormalities enable opportunities for future clinical implementation.

The results of this study provide a basis for future research. Screening for coronary artery calcification could aid in patient-specific and early risk assessment. Ultimately, earlier diagnostics and preventative measures could be initiated. Trauma patients might benefit from opportunistic screening using routine trauma CT images. However, this potential requires further study, and external validation of our findings in a comparable trauma population is needed. Furthermore, adding patient-specific risk factors to prediction models and performance analyses could result in quality improvement. Also, it could improve benchmark analysis across medical centers and medical systems.

In addition to a basis for future research, it could be interesting to assess other cardiovascular abnormalities visible on CT imaging, such as calcifications in the supra-aortic and iliac arteries. These predictors also could illustrate a worse pre-injury condition. Moreover, determining a patient-specific condition might be predictive for complications we did not investigate, such as arrhythmias, electrolyte disorders, and respiratory failure.

There were several limitations to the current study. Firstly, the models might have been overfitted due to the small number of events in the study cohort, possibly resulting in overestimating or underestimating the actual effect sizes. Furthermore, the small number of events in this study population could limit the power of our study; no (post hoc) power analysis was conducted. Secondly, coronary calcifications were scored on CT images performed according to our trauma protocol instead of electrocardiogram-guided, which is requisite in actual diagnostics. The routine imaging mode attenuates the possibility of detecting small calcifications in the coronary arteries or grading stenosis severity; however, the study situation reflects clinical practice in the trauma setting and is not intended for dedicated coronary artery disease diagnosis. Thirdly, we did not correct for competing risks using a statistical method, such as Fine and Gray modeling. Competing risks impede the occurrence of the (primary) outcome. The main competing risk in our study was death, which was part of an adverse discharge condition. We hypothesize that deceased patients were in a worse clinical state before dying than those alive were before being discharged; the development of a complication would be more likely around death than discharge. Hence, the eight deaths among 433 patients with 93 complications might have resulted in a small underestimation of the actual effect size. Lastly, patients could be included with first CT imaging up to seven days after admission. This criterion makes it harder to select the exact moment that opportunistic screening can be performed for risk assessment, and the indication for imaging will be different for early and later CT scans. However, as roughly 100% of the analyzed images were made within one day of admission, the results remain directive for early risk assessment. Furthermore, the results and conclusions were similar when only patients with a CT on the day of admission were included in the analyses (unpublished data).

In conclusion, left main coronary artery calcification visible on routine CT imaging in level-1 trauma patients was independently associated with in-hospital complications. Furthermore, increasing multivessel coronary artery calcification visible on CT is significantly associated with an adverse discharge condition. The findings of this study may help to identify trauma patients quickly and objectively at risk for complications in an early stage without performing additional diagnostics or interventions.

## Supplementary Information

Below is the link to the electronic supplementary material.

Supplementary file1 (DOCX 41 KB)

## Data Availability

The study data will not be made available.

## Abbreviations

AIS: Abbreviated Injury Scale
ASA: American Society of Anesthesiologists
CCI: Charlson Comorbidity Index
CI: Confidence interval
CT: Computed tomography
GCS: Glasgow Coma Scale
GOS: Glasgow Outcome Scale
HLOS: Hospital length of stay
ICU: Intensive care unit
IQR: Interquartile range
ISS: Injury Severity Score
LAD: Left anterior descending coronary artery
LCx: Left circumflex coronary artery
LM: Left main coronary artery
OR: Odds ratio
RCA: Right coronary artery
SD: Standard deviation
UMCU: University Medical Center Utrecht
