# Supplementary material for: Cardiovascular parameters on computed tomography are independently associated with in-hospital complications and outcomes in level-1 trauma patients
Source: Eur J Trauma Emerg Surg. 2022 Nov 27;49(3):1295–302. doi: 10.1007/s00068-022-02168-7 (PMC10229702; doi:10.1007/s00068-022-02168-7)
Supplement: Supplementary file 1 — Supplementary file1 (DOCX 41 KB) [file 68_2022_2168_MOESM1_ESM.docx]

| **Supplementary Table 1.** Cohen’s kappas (κ) for interobserver variability in scoring the radiologic abnormalities. | |
| --- | --- |
| **Radiologic parameter** | **κ** |
| Coronary arteries |  |
| Left main | 0.88 |
| Left anterior descending | 0.80 |
| Circumflex | 0.80 |
| Right coronary artery | 0.81 |
| Abdominal aorta | 0.79 |
| Pulmonary emphysema | 0.77 |

| Supplemental Table 2. Characteristics of and differences between subgroups of trauma patients who underwent CT imaging of the thorax or abdomen in a level-1 trauma center (n = 433). Stratified by infectious complications and delirium. | | | | | | | |
| --- | --- | --- | --- | --- | --- | --- | --- |
| Variable | **No infectious complication**  **(n = 367)** | **Infectious complication**  **(n = 66)** | ***p* value** |  | **No delirium**  **(n = 414)** | **Delirium**  **(n = 19)** | ***p* value** |
| Age (median [IQR]) | 48 [29 – 63] | 56 [45 – 72] | 0.001 |  | 49 [29 – 63] | 73 [66 – 87] | <0.001 |
| Sex |  |  | 0.442 |  |  |  | 0.699 |
| Male | 118 (32.2%) | 25 (37.9%) |  |  | 138 (33.3%) | 5 (26.3%) |  |
| Female | 249 (67.8%) | 41 (62.1%) |  |  | 276 (66.7%) | 14 (73.7%) |  |
| ASA classification (median [IQR]) | 2 [1 – 2] |  | 0.201 |  |  |  | 0.082 |
| 1 – 2 | 329 (89.6%) | 55 (83.3%) |  |  | 370 (89.4%) | 14 (73.7%) |  |
| 3 – 4 | 38 (10.4%) | 11 (16.7%) |  |  | 44 (10.6%) | 5 (26.3%) |  |
| Mechanism of injury |  |  | 0.556 |  |  |  | 0.777 |
| MVC | 130 (35.4%) | 23 (34.8%) |  |  | 148 (35.7%) | 5 (26.3%) |  |
| Bicycle | 52 (14.2%) | 14 (21.2%) |  |  | 63 (15.2%) | 3 (15.8%) |  |
| High-energetic fall | 54 (14.7%) | 13 (19.7%) |  |  | 64 (15.5%) | 3 (15.8%) |  |
| Low-energetic fall | 84 (22.9%) | 10 (15.2%) |  |  | 90 (21.7%) | 4 (21.1%) |  |
| Other | 47 (12.8%) | 6 (9.1%) |  |  | 49 (11.8%) | 4 (21.1%) |  |
| ISS (median [IQR]) | 9 [5 – 14] | 18 [13 – 25] | <0.001 |  | 9 [5 – 14] | 14 [12 – 18] | 0.007 |
| <16 | 302 (82.3%) | 25 (37.9%) |  |  | 315 (76.1%) | 12 (63.2%) |  |
| 16 – 25 | 50 (13.6%) | 25 (37.9%) |  |  | 69 (16.7%) | 6 (31.6%) |  |
| >25 | 15 (4.1%) | 16 (24.2%) |  |  | 30 (7.2%) | 1 (5.3%) |  |
| GCS score (median [IQR]) | 15 [14 – 15] | 15 [14 – 15] | 0.509 |  | 15 [14 – 15] | 15 [14 – 15] | 0.420 |
| AIS score per body region  (median [IQR]) |  |  |  |  |  |  |  |
| Head | 1 [0 – 1] | 0 [0 – 1] | 0.765 |  | 0 [0 – 1] | 1 [0 – 1] | 0.067 |
| Thorax | 0 [0 – 3] | 3 [0 – 3] | <0.001 |  | 1 [0 – 3] | 2 [0 – 3] | 0.368 |
| Abdomen | 0 [0 – 0] | 0 [0 – 2] | <0.001 |  | 0 [0 – 0] | 0 [0 – 0] | 0.601 |
| Spine | 0 [0 – 2] | 2 [0 – 2] | <0.001 |  | 0 [0 – 2] | 2 [0 – 3] | 0.008 |
| Upper extremity | 0 [0 – 1] | 0 [0 – 2] | 0.012 |  | 0 [0 – 1] | 0 [0 – 2] | 0.382 |
| Lower extremity | 0 [0 – 1] | 1 [0 – 3] | <0.001 |  | 0 [0 – 1] | 1 [0 – 2] | 0.169 |
| Abbreviations: AIS, Abbreviated Injury Scale; ASA, American Society of Anesthesiologists; GCS, Glasgow Coma Scale; IQR, interquartile range; ISS, Injury Severity Score; MVC, motor vehicle collision. | | | | | | | |

| **Supplementary Table 3.** The association of scored radiologic parameters and infectious complications in trauma patients who underwent CT imaging of the thorax or abdomen in a level-1 trauma center, before and after adjustment for covariates. Logistic regression analysis was used. | | | | | | | | |
| --- | --- | --- | --- | --- | --- | --- | --- | --- |
|  |  |  |  | **Crude** | |  | **Adjusted^a^** | |
| **Variable** | **Score** | **No infectious complication**  **(n = 367)** | **Infectious complication**  **(n = 66)** | **OR (95% CI)** | ***p* value** |  | **OR (95% CI)** | ***p* value** |
| **Left main** | 0 | 326 | 49 | reference | |  | reference | |
|  | 1 | 26 | 16 | 4.09 (2.02 — 8.12) | <0.001 |  | 3.86 (1.62 — 9.24) | <0.001 |
|  | Missing | 15 | 1 |  |  |  |  |  |
| **Number of calcified coronary arteries** | 0 | 255 | 39 | reference | |  | reference | |
|  | 1 | 48 | 6 | 0.82 (0.30 — 1.91) | 0.67 |  | 0.56 (0.17 — 1.63) | 0.32 |
|  | 2 | 23 | 9 | 2.56 (1.06 — 5.78) | 0.03 |  | 1.53 (0.49 — 4.55) | 0.45 |
|  | 3 | 26 | 11 | 2.77 (1.23 — 5.93) | 0.01 |  | 1.67 (0.59 — 4.63) | 0.32 |
|  | Missing | 15 | 1 | *p* for trend^b^ | 0.003 |  | *p* for trend^c^ | 0.29 |
| **Abdominal aorta** | 0 | 202 | 26 | reference | |  | reference | |
|  | 1 | 65 | 11 | 1.31 (0.59 — 2.75) | 0.48 |  | 0.89 (0.31 — 2.41) | 0.82 |
|  | 2 | 36 | 8 | 1.73 (0.69 — 3.97) | 0.22 |  | 0.71 (0.20 — 2.36) | 0.58 |
|  | 3 | 20 | 5 | 1.94 (0.61 — 5.29) | 0.22 |  | 1.07 (0.23 — 4.44) | 0.93 |
|  | 4 | 34 | 16 | 3.66 (1.76 — 7.49) | <0.001 |  | 1.64 (0.48 — 5.71) | 0.43 |
|  | Missing | 10 | 0 | *p* for trend^b^ | <0.001 |  | *p* for trend^c^ | 0.68 |
| **Pulmonary emphysema** | 0 | 331 | 54 | reference | |  | reference | |
|  | 1 | 24 | 10 | 2.55 (1.11 — 5.50) | 0.02 |  | 1.97 (0.73 — 4.97) | 0.16 |
|  | Missing | 12 | 2 |  |  |  |  |  |
| ^a^Separately adjusted for age, gender, ASA classification, and ISS; ^b^using the Cochran-Armitage test; ^c^using the likelihood ratio test in model with and without the variable. | | | | | | | | |
| **Abbreviations:** ASA, American Society of Anesthesiologists; CT, computed tomography; CI, confidence interval; ISS, Injury Severity Score; OR, odds ratio. | | | | | | | | |

| **Supplementary Table 4.** The association of scored radiologic parameters and pneumonia in trauma patients who underwent CT imaging of the thorax or abdomen in a level-1 trauma center, before and after adjustment for covariates. Logistic regression analysis was used. | | | | | | | | |
| --- | --- | --- | --- | --- | --- | --- | --- | --- |
|  |  |  |  | **Crude** | |  | **Adjusted^a^** | |
| **Variable** | **Score** | **No pneumonia**  **(n = 394)** | **Pneumonia**  **(n = 39)** | **OR (95% CI)** | ***p* value** |  | **OR (95% CI)** | ***p* value** |
| **Left main** | 0 | 345 | 30 | reference | |  | reference | |
|  | 1 | 34 | 8 | 2.71 (1.09 — 6.14) | 0.02 |  | 2.27 (0.78 — 6.30) | 0.12 |
|  | Missing | 15 | 1 |  |  |  |  |  |
| **Number of calcified coronary arteries** | 0 | 271 | 23 | reference | |  | reference | |
|  | 1 | 51 | 3 | 0.69 (0.16 — 2.09) | 0.56 |  | 0.43 (0.07 — 1.76) | 0.29 |
|  | 2 | 26 | 6 | 2.72 (0.94 — 6.94) | 0.05 |  | 1.70 (0.42 — 6.15) | 0.43 |
|  | 3 | 31 | 6 | 2.28 (0.79 — 5.73) | 0.10 |  | 1.29 (0.36 — 4.27) | 0.68 |
|  | Missing | 15 | 1 | *p* for trend^b^ | 0.03 |  | *p* for trend^c^ | 0.42 |
| **Abdominal aorta** | 0 | 211 | 17 | reference | |  | reference | |
|  | 1 | 72 | 4 | 0.69 (0.19 — 1.94) | 0.52 |  | 0.33 (0.07 — 1.32) | 0.14 |
|  | 2 | 38 | 6 | 1.96 (0.67 — 5.06) | 0.18 |  | 0.64 (0.14 — 2.62) | 0.55 |
|  | 3 | 22 | 3 | 1.69 (0.37 — 5.55) | 0.43 |  | 0.73 (0.11 — 4.07) | 0.73 |
|  | 4 | 41 | 9 | 2.72 (1.09 — 6.42) | 0.02 |  | 1.00 (0.23 — 4.43) | 1.00 |
|  | Missing | 10 | 0 | *p* for trend^b^ | 0.01 |  | *p* for trend^c^ | 0.48 |
| **Pulmonary emphysema** | 0 | 354 | 31 | reference | |  | reference | |
|  | 1 | 28 | 6 | 2.45 (0.86 — 6.02) | 0.07 |  | 1.63 (0.45 — 4.94) | 0.42 |
|  | Missing | 12 | 2 |  |  |  |  |  |
| ^a^Separately adjusted for age, gender, ASA classification, and ISS; ^b^using the Cochran-Armitage test; ^c^using the likelihood ratio test in model with and without the variable. | | | | | | | | |
| **Abbreviations:** ASA, American Society of Anesthesiologists; CT, computed tomography; CI, confidence interval; ISS, Injury Severity Score; OR, odds ratio. | | | | | | | | |

| **Supplementary Table 5.** The association of scored radiologic parameters and delirium in trauma patients who underwent CT imaging of the thorax or abdomen in a level-1 trauma center, before and after adjustment for covariates. Logistic regression analysis was used. | | | | | | | | |
| --- | --- | --- | --- | --- | --- | --- | --- | --- |
|  |  |  |  | **Crude** | |  | **Adjusted^a^** | |
| **Variable** | **Score** | **No delirium**  **(n = 414)** | **Delirium**  **(n = 19)** | **OR (95% CI)** | ***p* value** |  | **OR (95% CI)** | ***p* value** |
| **Left main** | 0 | 366 | 9 | reference | |  | reference | |
|  | 1 | 35 | 7 | 8.13 (2.76 — 23.18) | <0.001 |  | 3.34 (1.00 — 11.05) | 0.05 |
|  | Missing | 13 | 3 |  |  |  |  |  |
| **Number of calcified coronary arteries** | 0 | 289 | 5 | reference | |  | reference | |
|  | 1 | 52 | 2 | 2.22 (0.31 — 10.62) | 0.35 |  | 0.68 (0.09 — 3.71) | 0.67 |
|  | 2 | 28 | 4 | 8.26 (1.95 — 32.97) | <0.001 |  | 1.81 (0.35 — 9.22) | 0.47 |
|  | 3 | 32 | 5 | 9.03 (2.4 — 34.12) | <0.001 |  | 1.66 (0.33 — 8.39) | 0.53 |
|  | Missing | 13 | 3 | *p* for trend^b^ | <0.001 |  | *p* for trend^c^ | 0.66 |
| **Abdominal aorta** | 0 | 227 | 1 | reference | |  | reference | |
|  | 1 | 72 | 4 | 12.61 (1.83 — 248.83) | 0.02 |  | 3.99 (0.50 — 83.89) | 0.24 |
|  | 2 | 41 | 3 | 16.61 (2.07 — 340.30) | 0.02 |  | 2.69 (0.27 — 62.48) | 0.44 |
|  | 3 | 23 | 2 | 19.74 (1.82 — 434.47) | 0.02 |  | 2.43 (0.16 — 67.58) | 0.53 |
|  | 4 | 41 | 9 | 49.83 (9.02 — 931.30) | <0.001 |  | 7.25 (0.81 — 169.48) | 0.12 |
|  | Missing | 10 | 0 | *p* for trend^b^ | <0.001 |  | *p* for trend^c^ | 0.36 |
| **Pulmonary emphysema** | 0 | 369 | 16 | reference | |  | reference | |
|  | 1 | 32 | 2 | 1.44 (0.22 — 5.37) | 0.64 |  | 0.82 (0.12 — 3.41) | 0.81 |
|  | Missing | 13 | 1 |  |  |  |  |  |
| ^a^Separately adjusted for age, gender, ASA classification, and ISS; ^b^using the Cochran-Armitage test; ^c^using the likelihood ratio test in model with and without the variable. | | | | | | | | |
| **Abbreviations:** ASA, American Society of Anesthesiologists; CT, computed tomography; CI, confidence interval; ISS, Injury Severity Score; OR, odds ratio. | | | | | | | | |

| **Supplementary Table 6.** The association of scored radiologic parameters and discharge condition, measured in the GOS score, in trauma patients who underwent CT imaging of the thorax or abdomen in a level-1 trauma center, before and after adjustment for covariates. Logistic regression analysis was used. | | | | | | | | |
| --- | --- | --- | --- | --- | --- | --- | --- | --- |
|  |  |  |  | **Crude** | |  | **Adjusted^a^** | |
| **Variable** | **Score** | **No adverse GOS**  **(n = 406)** | **Adverse GOS**  **(n = 27)** | **OR (95% CI)** | ***p* value** |  | **OR (95% CI)** | ***p* value** |
| **Left main** | 0 | 352 | 23 | reference | |  | reference | |
|  | 1 | 39 | 3 | 1.18 (0.27 — 3.58) | 0.80 |  | 0.86 (0.17 — 3.23) | 0.84 |
|  | Missing | 15 | 1 |  |  |  |  |  |
| **Number of calcified coronary arteries** | 0 | 280 | 14 | reference | |  | reference | |
|  | 1 | 53 | 1 | 0.38 (0.02 — 1.94) | 0.35 |  | 0.14 (0.00 — 1.71) | 0.22 |
|  | 2 | 26 | 6 | 4.62 (1.53 — 12.6) | <0.001 |  | 5.18 (1.14 — 23.44) | 0.03 |
|  | 3 | 32 | 5 | 3.13 (0.96 — 8.78) | 0.04 |  | 3.03 (0.70 — 12.81) | 0.13 |
|  | Missing | 15 | 1 | *p* for trend^b^ | 0.004 |  | *p* for trend^c^ | 0.02 |
| **Abdominal aorta** | 0 | 219 | 9 | reference | |  | reference | |
|  | 1 | 69 | 7 | 2.47 (0.85 — 6.87) | 0.08 |  | 2.41 (0.57 — 10.62) | 0.23 |
|  | 2 | 40 | 4 | 2.43 (0.63 — 7.87) | 0.15 |  | 1.06 (0.14 — 6.82) | 0.95 |
|  | 3 | 22 | 3 | 3.32 (0.70 — 12.10) | 0.09 |  | 3.16 (0.38 — 24.54) | 0.27 |
|  | 4 | 46 | 4 | 2.12 (0.55 — 6.80) | 0.23 |  | 1.21 (0.17 — 8.67) | 0.84 |
|  | Missing | 10 | 0 | *p* for trend^b^ | 0.09 |  | *p* for trend^c^ | 0.58 |
| **Pulmonary emphysema** | 0 | 364 | 21 | reference | |  | reference | |
|  | 1 | 30 | 4 | 2.31 (0.64 — 6.56) | 0.15 |  | 1.29 (0.24 — 4.88) | 0.74 |
|  | Missing | 12 | 2 |  |  |  |  |  |
| ^a^Separately adjusted for age, gender, ASA classification, and ISS; ^b^using the Cochran-Armitage test; ^c^using the likelihood ratio test in model with and without the variable. | | | | | | | | |
| **Abbreviations:** ASA, American Society of Anesthesiologists; CT, computed tomography; CI, confidence interval; ISS, Injury Severity Score; OR, odds ratio. | | | | | | | | |

| **Supplementary Table 7.** The association of scored radiologic parameters and hospital length of stay in trauma patients who underwent CT imaging of the thorax or abdomen in a level-1 trauma center, before and after adjustment for covariates. Linear regression analysis was used. | | | | | | | |
| --- | --- | --- | --- | --- | --- | --- | --- |
|  |  |  | **Crude** | |  | **Adjusted^a^** | |
| **Variable** | **Score** | **Variable present in patients** | **β-coefficient (95% CI)** | ***p* value** |  | **β-coefficient (95% CI)** | ***p* value** |
| **Left main** | 0 | 375 | reference | |  | reference | |
|  | 1 | 42 | 1.71 (1.29 — 2.25) | <0.001 |  | 1.26 (0.99 — 1.61) | 0.07 |
|  | Missing | 16 |  |  |  |  |  |
| **Number of calcified coronary arteries** | 0 | 294 | reference | |  | reference | |
|  | 1 | 54 | 1.07 (0.78 — 1.45) | 0.68 |  | 0.87 (0.66 — 1.14) | 0.30 |
|  | 2 | 32 | 1.49 (1.03 — 2.14) | 0.03 |  | 1.17 (0.85 — 1.62) | 0.33 |
|  | 3 | 37 | 1.78 (1.28 — 2.47) | <0.001 |  | 1.22 (0.90 — 1.65) | 0.21 |
|  | Missing | 16 | *p* for trend^b^ | <0.001 |  | *p* for trend^c^ | 0.11 |
| **Abdominal aorta** | 0 | 228 | reference | |  | reference | |
|  | 1 | 76 | 1.10 (0.85 — 1.42) | 0.48 |  | 0.85 (0.67 — 1.09) | 0.20 |
|  | 2 | 44 | 1.43 (1.03 — 1.97) | 0.03 |  | 0.87 (0.64 — 1.18) | 0.36 |
|  | 3 | 25 | 1.37 (0.91 — 2.07) | 0.14 |  | 0.86 (0.58 — 1.29) | 0.46 |
|  | 4 | 50 | 2.01 (1.48 — 2.73) | <0.001 |  | 1.17 (0.83 — 1.64) | 0.38 |
|  | Missing | 10 | *p* for trend^b^ | <0.001 |  | *p* for trend^c^ | 0.18 |
| **Pulmonary emphysema** | 0 | 385 | reference | |  | reference | |
|  | 1 | 34 | 1.17 (0.82 — 1.67) | 0.40 |  | 0.89 (0.67 — 1.18) | 0.42 |
|  | Missing | 14 |  |  |  |  |  |
| ^a^Separately adjusted for age, gender, ASA classification, and ISS; ^b^using the Cochran-Armitage test; ^c^using the likelihood ratio test in model with and without the variable. | | | | | | | |
| **Abbreviations:** ASA, American Society of Anesthesiologists; CT, computed tomography; CI, confidence interval; ISS, Injury Severity Score; OR, odds ratio. | | | | | | | |
